# The effect of renal function change on renal cell carcinoma patients with tumor thrombus after nephrectomy and thrombectomy: a large Chinese center experience

**DOI:** 10.1186/s12885-020-6563-7

**Published:** 2020-01-28

**Authors:** Zhuo Liu, Shiying Tang, Xiaojun Tian, Hongxian Zhang, Guoliang Wang, Shudong Zhang, Lulin Ma

**Affiliations:** Department of Urology, Peking University Third Hospital, 49 North Garden Rd., Haidian District, Beijing, People’s Republic of China

**Keywords:** Renal cell carcinoma with tumor thrombus, Postoperative renal function, Absolute change in renal function, Percent change in renal function

## Abstract

**Background:**

To explore the influencing factors of perioperative renal function change and their relationship with prognosis on renal cell carcinoma (RCC) patients with tumor thrombus after nephrectomy and thrombectomy.

**Methods:**

The clinical and pathological data of 135 patients with RCC and tumor thrombus, who underwent nephrectomy and thrombectomy at Peking University Third Hospital from May 2015 to July 2018, was retrospectively analyzed. Absolute change in estimated glomerular filtration rate (eGFR) (ACE) and percent change in eGFR (PCE) were calculated by preoperative and postoperative renal function. Linear regression analysis was used to explore the influencing factors of ACE and PCE, and logistic regression analysis was used to explore the influencing factors of worse postoperative renal function [eGFR≤60 mL/(min × 1.73 m^2)]. Cancer-specific survival (CSS) was estimated by Kaplan-Meier method and multivariate Cox regression, which were used to explore the effect of ACE and PCE on prognosis.

**Results:**

Of all the 135 patients, 101 patients (74.8%) were male and 34 patients (25.2%) were female. The mean preoperative eGFR was 73.9 ± 21.8 mL/(min × 1.73 m^2) and postoperative eGFR was 69.5 ± 25.2 mL/(min × 1.73 m^2). In multivariate linear regression analysis, preoperative eGFR (*P* < 0.001) and pathological type (*P* = 0.038) were significant predictive factors of ACE. In aspect of PCE, preoperative eGFR (P < 0.001) and pathological type (*P* = 0.002) were significant predictors. In multivariate logistic regression analysis, preoperative eGFR (*P* = 0.016) was the only risk factor of predicting worse postoperative renal function. During follow-up, 22 patients (16.3%) were dead due to RCC. According to ROC analysis, the cut off value of ACE and PCE was 13.9 and 0.16, respectively. ACE> 13.9 and PCE > 0.16 indicated worse CSS (*P* = 0.006 and *P* = 0.047, respectively). However, in multivariate Cox regression analysis of several related factors, perinephric tissues invasion (*P* = 0.001), sarcomatoid differentiation (P = 0.001) and ACE> 13.9 (*P* = 0.002) were significant prognostic factors for CSS. PCE > 0.16 seemed to be not (*P* = 0.055).

**Conclusion:**

We explored several clinicopathological risk factors of predicting renal function change and their relationship with prognosis of RCC patients with tumor thrombus after nephrectomy and thrombectomy. The renal function change, which was associated with preoperative eGFR and pathological type, was prognostic risk factor for CSS and ACE> 13.9 indicated the worse prognosis.

## Background

Renal cell carcinoma (RCC) represents 2–3% of all cancers [1], and this data was 2.2% in China [2]. The incidence of RCC has increased approximately 5% of new cancers in the USA [3] and ascended about 2% steadily in European union over the past decade [4]. Moreover, of all the patients with RCC, approximate 4–10% have vein invasion, including the renal vein or/and the inferior vena cava (IVC) which could even extend up to the right atrium [1]. For these patients, radical nephrectomy (RN) with thrombectomy appears to be the gold standard of treatment gradually, which offers the potential cure with a 5-year cancer-specific survival (CSS) of 40–65% [1]. Furthermore, patients following cytoreductive nephrectomy (CN) have a longer overall survival (OS) compared with those without CN (23.9 vs. 9 months, *P* < 0.001) [5].

Although there are several effective treatments for this malignant tumor, patients are still at a certain risk of reduction in renal function due to loss of renal tissue after performing RN with thrombectomy [6]. Zabor et al. [6] explored the long-term renal function recovery after RN and demonstrated that only 45% of patients had renal function recovery in 2 years. Several models have been made to predict the probability of renal function recovery in patients following pure RN [7, 8]. 2017 AUA guidelines also recommended that it was necessary for clinicians to consider renal function outcomes when making management process [9]. In previous multicenter studies of RCC patients after RN, old age, low preoperative estimated glomerular filtration rate (eGFR) and high comorbidity are the significant predictive factors of poor postoperative renal function [10–12].

However, to our knowledge, there was less research about evaluating the effect of perioperative renal function change in RCC patients with tumor thrombus (TT). Thus, while trying to validate the previous conclusion in pure RN, our aim is to explore the influencing factors of perioperative renal function change and their relationship with prognosis on RCC patients with TT after nephrectomy and thrombectomy in a large Chinese center.

## Methods

From May 2015 to July 2018, 135 patients with RCC with TT who underwent RN or CN and thrombectomy at Peking University Third Hospital was retrospectively analyzed. All the patients underwent routine blood examinations, chest and abdominal computed tomography (CT) or magnetic resonance imaging (MRI), and/or bone scans preoperatively. Before surgery, a multidisciplinary team, including specialists from urology, general surgery, cardiac surgery, anesthesiology, and radiology departments, gives a comprehensive assessment of the patient.

Demographic and clinicopathological data were evaluated. The level of TT was classified according to the Mayo classification [13]. Renal function was assessed by eGFR, which was based on serum creatinine calculated by the Modification of Diet in Renal Disease (MDRD) [14] in one week preoperatively and 1 month postoperatively. The change of perioperative renal function was presented by absolute change in eGFR (ACE) and percent change in eGFR (PCE), which were proposed by Haifler et al. ACE = eGFR_postoperative_ - eGFR_preoperative_ and PCE = (eGFR_postoperative_ - eGFR_preoperative_) / eGFR_preoperative_, respectively [15]. The American Society of Anesthesiologists classification was used to classify physical condition and surgical risk [16]. The postoperative complications were evaluated by modified Clavien grading system [17] and grade higher than III was considered to be a serious complication. The postoperative specimens were evaluated by two experienced pathologists in our institution. Pathological features including histology and tumor grade were also reviewed according to the 2016 World Health Organization (WHO) classification [18].

Cytoreductive nephrectomy refers to the patients with possible metastasis found by imaging before operation. For the metastasis renal cell carcinoma and tumor thrombus patients with good condition who can tolerate operation, we also suggest them operation treatment. Because the growth rate of tumor thrombus usually grows faster, which may cause sudden cardiac death caused by atrial cancer thrombus. Appropriate treatments, such as immunotherapy and targeted therapy, were provided in cases of local recurrence or distant metastasis in our study. Follow-up, including laboratory data, chest radiography, urinary ultrasonography, and enhanced urinary CT/MRI, was performed every 3 months in the first year, every 6 months until year 5, and annually thereafter. The co-primary endpoints of the study were cancer-specific death, and all-cause mortality.

Continuous variables were presented as the mean value ± SD and were analyzed using Mann-Whitney U test. Categorical variables were compared by using the Pearson x^2^ test. Linear regression analysis was used to explore the risk factors of ACE and PCE, and logistic regression analysis was used to explore the predictors of postoperative renal insufficiency [eGFR≤60 mL/(min × 1.73 m^2)]. We made multivariate analysis for factors which had statistical significance in univariate analysis. The significant factors of univariate analysis were CSS was estimated by the Kaplan-Meier method and compared by log-rank test in different level of ACE and PCE, which was calculated by Receiver Operating Characteristic (ROC) curve. A two-sided *P* value < 0.05 was considered to be statistically significant. All data were collected and analyzed by SPSS 22.0 software (IBM Corp, Armonk, NY, USA).

## Results

Demographic and clinicopathological characteristics of 135 patients with RCC with TT, including 101 (74.8%) males and 34 (25.2%) females, are summarized in Table 1. The mean of preoperative eGFR was 73.9 ± 21.8 mL/(min × 1.73 m^2) and postoperative eGFR was 69.5 ± 25.2 mL/(min × 1.73 m^2). The postoperative eGFR of 42 patients (31.1%) was below 60 mL/(min × 1.73 m^2) and 93 patients (68.9%) had adequate renal function [> 60 mL/(min × 1.73 m^2)]. There were 31 (23.0%) patients with renal vein tumor thrombus and 104 (77.0%) with IVC tumor thrombus including 38 with level I, 39 with level II, 16 with level III and 11 with level IV.

**Table 1:** Patient demographic and postoperative data

Paprameters	n / x ± s
Gender	
Male	101
Female	34
Age, years	59.2 ± 10.8
^Body mass index, kg/m^2^	23.5 ± 3.6
ASA classification	
1	9
2	102
3	24
Side	
Left	49
Right	86
Tumor diameter, cm	8.8 ± 3.5
Tumor thrombus grade	
0	31
I	38
II	39
III	16
IV	11
Preoperative serum creatinine, µmol/L	101.9 ± 78.2
Postoperative serum creatinine, µmol/L	123.1 ± 121.6
Preoperative eGFR, mL/(min × 1.73 m^2)	73.9 ± 21.8
Postoperative eGFR, mL/(min × 1.73 m^2)	69.5 ± 25.2
Operation approach	
Laparoscopic approach	74
Open approach	61
Ipsilateral adrenalectomy	
No	59
Yes	76
T stage	
T3a	27
T3b	83
T3c	7
T4	18
Lymph node dissection	
No	65
Yes	70
Vascular wall invasion	
No	82
Yes	53
Operation time, min	337.0 ± 122.7
Intraoperative hemorrhage, ml	1387.7 ± 1640.6
Pathological type	
Clear cell carcinoma	115
Non clear cell carcinoma	20
WHO/ISUP 2016 Nuclear classification	
1	3
2	47
3	51
4	34
Lymph node metastasis	
No	125
Yes	10
Metastasis or invasion of adrenal glands	
No	117
Yes	18
Metastasis	
No	100
Yes	35
Lymphatic vascular invasion	
No	106
Yes	29
Perinephric tissues invasion	
No	94
Yes	41
Renal pelvis invasion	
No	105
Yes	30
Necrosis	
No	66
Yes	69
Sarcomatoid differentiation	
No	113
Yes	22
Postoperative complications	
No	79
Yes	56
Serious complications	
No	38
Yes	18

*eGFR* estimated glomerular filtration rate, *ISUP* International Society of Urological Pathology, *ASA* American Society of Anesthesiologists

All the patients underwent RN or CN with thrombectomy successfully. There are four patients with thoracotomy and extracorporeal circulation; two patients with thoracotomy without extracorporeal circulation; others without thoracotomy. During operation, It is common to clam the contralateral renal vein. Only one patients underwent venal graft placement. During the operation, the distal end of inferior vena cava was found to be free of tumor invasion. A part of the uninjured wall of inferior vena cava was resected and reconstructed as the distal end of renal vein in the healthy side to ensure renal blood flow in the healthy side.

For operation-related data, the mean operation time was 337.0 ± 122.7 min and the mean intraoperative blood loss 1387.7 ± 1640.6 ml. Fifty six patients had postoperative complications according to Clavien classification, while only 18 patients had serious complications. In all patients, 115 patients had clear cell carcinoma and 20 patients were other histological types. The median follow-up was 11.4 months (range: 0–37). During follow-up, 22 patients (16.3%) were dead due to the disease. The 1-year CSS of patients with low preoperative renal function is 87.5% and 2-year CSS of these is 58.3%. The 1-year CSS of patients with low postoperative renal function is 84.9% and 2-year CSS of these is 79.2%. The 3-year CSS of all the patients is 91.5% and 5-year CSS of these is 86.0%.

**Fig. 1:**
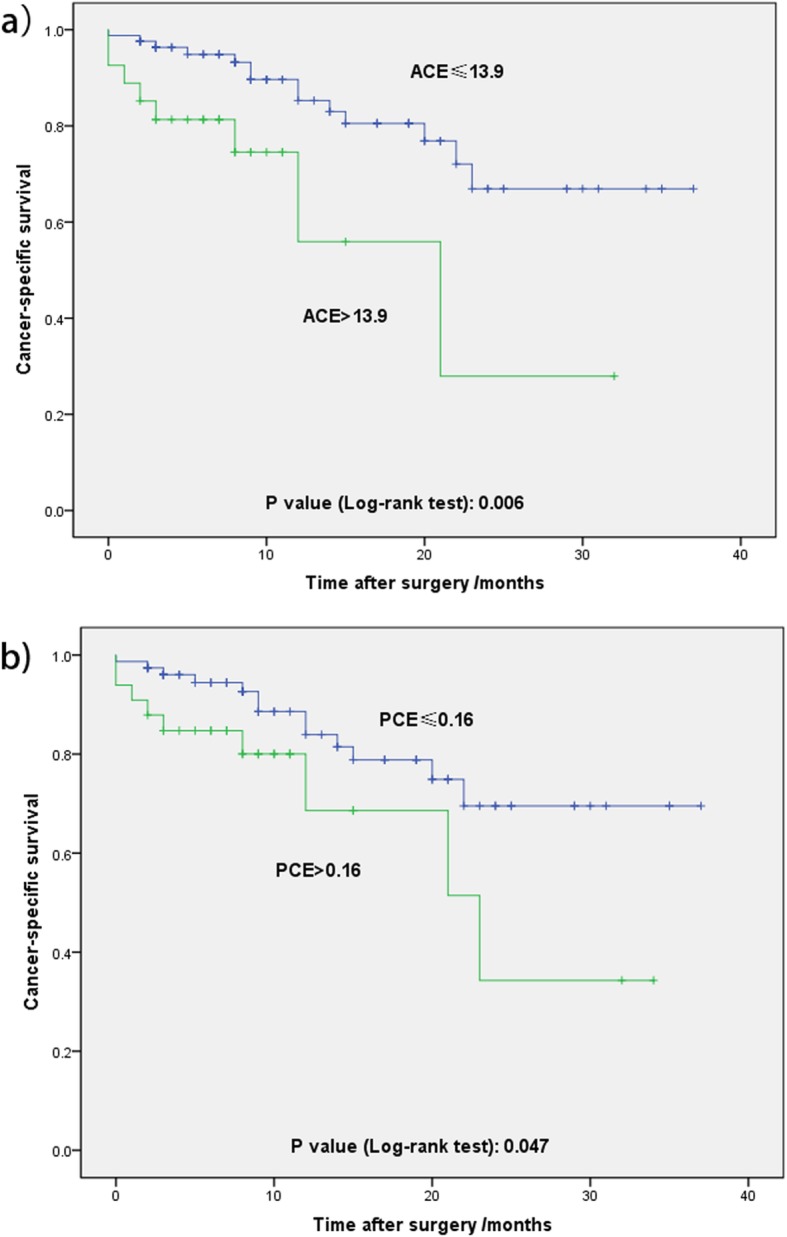
**a**) Kaplan-Meier survival curves for cancer-specific survival for the cut-off value of ACE. **b**) Kaplan-Meier survival curves for cancer-specific survival for the cut-off value of PCE

In multivariate linear regression analysis, preoperative eGFR (*P* < 0.001) and pathological type (*P* = 0.038) were significant predictive factors of ACE (Table 2). In aspect of PCE, preoperative eGFR (P < 0.001) and pathological type (*P* = 0.002) were significant predictors (Table 3). In multivariate logistic regression analysis, preoperative eGFR (*P* = 0.016), was the only risk factor of predicting worse postoperative renal function defined as eGFR≤60 mL/(min × 1.73 m^2) (Table 4). According to ROC curve analysis, the cut off value of ACE and PCE was 13.9 and 0.16, respectively. ACE> 13.9 and PCE > 0.16 indicated worse CSS (*P* = 0.006 and *P* = 0.047, respectively) in Kaplan-Meier analysis (Fig. 1a & b). However, in multivariate Cox regression analysis of several related factors, perinephric tissues invasion (*P* = 0.001), sarcomatoid differentiation (P = 0.001) and ACE> 13.9 (*P* = 0.002) were significant prognostic factors for CSS. PCE > 0.16 seemed to be not (*P* = 0.055) (Table 5).

**Table 2:** Linear regression analysis of absolute change in eGFR

Variables	Univariate analysis			Multivariate analysis		
	B	95%CI	P	B	95%CI	P
Gender (female vs. male)	5.724	−3.977-15.426	0.245			
Age	−0.208	− 0.599-0.183	0.295			
Body mass index	0.105	−1.090-1.301	0.862			
ASA classification	−6.273	−15.458-2.912	0.179			
Side (right vs. left)	1.175	−7.626-9.975	0.792			
Tumor diameter	0.975	−0.228-2.178	0.111			
Tumor thrombus grade	0.521	−3.013-4.055	0.771			
Preoperative serum creatinine	0.079	0.027–0.132	0.003^*^	–	–	–
Preoperative eGFR	−0.476	−0.653--0.299	< 0.001^*^	−0.455	− 0.631--0.278	< 0.001^*^
Operation approach (open vs. laparoscopy)	2.11	−6.387-10.607	0.624			
Operation time	0.022	−0.013-0.056	0.213			
Pathological type (non-clear cell carcinoma vs. clear cell carcinoma)	14.612	2.964–26.260	0.014^*^	11.416	0.658–22.174	0.038^*^
Vascular wall invasion (present vs. absent)	6.462	−2.135-15.058	0.139			
Lymphatic vascular invasion (present vs. absent)	−4.479	−14.733-5.775	0.389			
Perinephric tissues invasion (present vs. absent)	−2.692	−11.876-6.491	0.563			
Renal pelvis invasion (present vs. absent)	7.211	−2.872-17.293	0.16			
Necrosis (present vs. absent)	−1.846	−10.338-6.646	0.668			
Sarcomatoid differentiation (present vs. absent)	−1.769	−13.193-9.655	0.76			

*eGFR* estimated glomerular filtration rate, *ISUP* International Society of Urological Pathology; *p<0.05

**Table 3:** Linear regression analysis of percent change in eGFR

Variables	Univariate analysis			Multivariate analysis		
	B	95%CI	P	B	95%CI	P
Gender (female vs. male)	0.047	−0.289-0.383	0.782			
Age	− 0.009	− 0.023-0.004	0.182			
Body mass index	0.028	−0.013-0.069	0.182			
ASA classification	−0.107	−0.426-0.212	0.507			
Side (right vs. left)	−0.167	−0.469-0.135	0.276			
Tumor diameter	0.044	0.003–0.085	0.037^*^	0.037	−0.001-0.074	0.058
Tumor thrombus grade	0.016	−0.106-0.138	0.794			
Preoperative serum creatinine	0.01	0.009–0.011	< 0.001^*^	–	–	–
Preoperative eGFR	−0.014	−0.020--0.007	< 0.001^*^	−0.013	− 0.019--0.007	< 0.001^*^
Operation approach (open vs. laparoscopy)	−0.101	− 0.394-0.192	0.496			
Operation time	0.001	0.000–0.002	0.094			
Pathological type (non-clear cell carcinoma vs. clear cell carcinoma)	0.59	0.191–0.988	0.004^*^	0.623	0.236–1.010	0.002^*^
Vascular wall invasion (present vs. absent)	0.247	−0.049-0.543	0.101			
Lymphatic vascular invasion (present vs. absent)	−0.128	−0.485-0.229	0.48			
Perinephric tissues invasion (present vs. absent)	0.156	−0.163-0.475	0.336			
Renal pelvis invasion (present vs. absent)	0.368	0.020–0.716	0.038^*^			
Necrosis (present vs. absent)	−0.16	−0.455-0.134	0.284			
Sarcomatoid differentiation (present vs. absent)	−0.066	−0.464-0.332	0.743			

*eGFR* estimated glomerular filtration rate, *ISUP* International Society of Urological Pathology, *ASA* American Society of Anesthesiologists; *p<0.05

**Table 4:** Univariate and multivariate Logistic regression analysis of clinical data and worse postoperative renal function [eGFR≤60 mL/(min × 1.73 m^2)]

Variables	Univariate analysis			Multivariate analysis		
	OR	95%CI	P	OR	95%CI	P
Gender (female vs. male)	1.108	0.390–3.147	0.847			
Age	1	0.962–1.040	0.999			
Body mass index	0.905	0.804–1.020	0.101			
ASA classification						
I	–	–	0.437			
II	0.25	0.030–2.081	0.2			
III	0.25	0.026–2.416	0.231			
Side (right vs. left)	0.817	0.325–2.050	0.666			
Tumor diameter	1.088	0.944–1.254	0.247			
Tumor thrombus grade						
0	–	–	0.232			
I	0.24	0.058–0.991	0.049^*^			
II	0.216	0.052–0.898	0.035^*^			
III	0.54	0.077–3.775	0.535			
IV	0.42	0.058–3.029	0.389			
Preoperative serum creatinine	0.966	0.938–0.994	0.019^*^	–	–	–
Preoperative eGFR	1.054	1.013–1.096	0.009^*^	1.049	1.009–1.092	0.016^*^
Operation approach (open vs. laparoscopy)	1.576	0.642–3.864	0.321			
Ipsilateral adrenalectomy (present vs. absent)	1.492	0.615–3.616	0.376			
Lymph node dissection (present vs. absent)	0.821	0.339–1.992	0.663			
Operation time	0.998	0.995–1.002	0.344			
Pathological type (non-clear cell carcinoma vs. clear cell carcinoma)	2.692	0.570–12.707	0.211			
WHO/ISUP 2016 Nuclear classification (12vs. 34)	2.277	0.937–5.534	0.069			
Lymph node metastasis (present vs. absent)	1.134	0.220–5.847	0.881			
Metastasis (present vs. absent)	2.897	0.789–10.627	0.109			
Vascular wall invasion (present vs. absent)	0.482	0.195–1.192	0.114			
Lymphatic vascular invasion (present vs. absent)	1.864	0.573–6.067	0.301			
Perinephric tissues invasion (present vs. absent)	0.776	0.302–1.991	0.598			
Renal pelvis invasion (present vs. absent)	1.152	0.378–3.505	0.804			
Necrosis (present vs. absent)	0.372	0.145–0.956	0.04^*^	0.384	0.145–1.015	0.054
Sarcomatoid differentiation (present vs. absent)	0.718	0.244–2.114	0.548			
Postoperative complications (present vs. absent)	0.851	0.352–2.056	0.72			

*eGFR* estimated glomerular filtration rate, *ISUP* International Society of Urological Pathology, *ASA* American Society of Anesthesiologists; *p<0.05

**Table 5:** Univariate and multivariate Cox regression analysis of CSS in patients with renal cell carcinoma and tumor thrombus after radical nephrectomy and thrombectomy

Variables	Univariate analysis			Multivariate analysis		
	OR	95%CI	P	OR	95%CI	P
Gender (female vs. male)	0.327	0.076–1.405	0.133			
Age	0.995	0.955–1.036	0.808			
Body mass index	0.974	0.867–1.093	0.651			
ASA classification						
I	–	–	0.3			
II	0.942	0.123–7.189	0.954			
III	1.996	0.238–16.772	0.524			
Side (right vs. left)	0.762	0.325–1.786	0.532			
Tumor diameter	1.088	0.978–1.210	0.122			
Tumor thrombus grade			0.944			
0	–	–	0.716			
I	0.474	0.138–1.621	0.234			
II	0.706	0.222–2.248	0.556			
III	1.172	0.342–4.012	0.801			
IV	0.732	0.152–3.527	0.697			
Preoperative serum creatinine	0.999	0.991–1.008	0.885			
Preoperative eGFR	1	0.979–1.020	0.973			
Operation approach (open vs. laparoscopy)	2.085	0.890–4.884	0.091			
Ipsilateral adrenalectomy (present vs. absent)	2.087	0.846–5.149	0.11			
Lymph node dissection (present vs. absent)	3.008	1.109–8.162	0.031^*^			
Operation time	1.002	0.999–1.005	0.174			
Pathological type (non-clear cell carcinoma vs. clear cell carcinoma)	1.996	0.767–5.037	0.159			
WHO/ISUP 2016 Nuclear classification(12vs.34)	3.843	1.135–13.012	0.031^*^			
T stage						
T3a						
T3b						
T3c						
T4						
Lymph node metastasis (present vs. absent)	4.166	1.522–11.402	0.005^*^			
Metastasis (present vs. absent)	2.04	0.870–4.782	0.101			
Vascular wall invasion (present vs. absent)	1.18	0.471–2.952	0.724			
Lymphatic vascular invasion (present vs. absent)	2.305	0.996–5.334	0.051			
Perinephric tissues invasion (present vs. absent)	4.029	1.709–9.497	0.001^*^	4.497	1.844–10.970	0.001^*^
Renal pelvis invasion (present vs. absent)	1.876	0.679–5.180	0.225			
Necrosis (present vs. absent)	1.704	0.729–3.981	0.219			
Sarcomatoid differentiation (present vs. absent)	3.417	1.431–8.160	0.006^*^	4.666	1.834–11.873	0.001^*^
Postoperative complications (present vs. absent)	2.499	1.016–6.146	0.046^*^	2.243	0.898–5.603	0.084
ACE (> 13.9 vs. ≤13.9)	3.274	1.331–8.052	0.01^*^	4.2	1.657–10.642	0.002^*^
PCE (> 0.16 vs. ≤0.16)	2.322	0.982–5.494	0.055			

*eGFR* estimated glomerular filtration rate, *ISUP* International Society of Urological Pathology, *ASA* American Society of Anesthesiologists, *ACE* absolute change in eGFR, *PCE* percent change in eGFR; *p<0.05

## Discussion

In RCC patients with TT, RN with thrombectomy combined with metachronous adjuvant therapy seems to be the most effective methods to better prognosis [1]. However, this surgical methods might have effect on renal function owing to the loss of renal tissue and targeted therapy also has a certain impact on serum creatinine in patients with advanced RCC [19]. In previous studies, several researches have been conducted to confirm the risk factors of postoperative renal function following pure RN [6, 20, 21], while the conclusions were still in controversy and lack of data of which after thrombectomy. Thus, in order to validate and consummate previous results, we selected these specified patients and explored the effect of renal function change after nephrectomy and thrombectomy in a large Chinese center.

In our study, we validated the serum creatinine increased and the eGFR decreased postoperatively, revealing that RN with thrombectomy might also have an effect on patients’ renal function which was associated with OS^22^. Moreover, we made multivariate logistic regression analysis and showed that preoperative eGFR was the only risk factors of predicting chronic kidney disease [eGFR≤60 mL/(min × 1.73 m^2)]. In addition, the ACE and PCE were also vital important variables in evaluating renal function [15]. The greater the change of eGFR, the more apparent the descending of renal function after operation. It was also showed that among all the significant predictive factors, only the preoperative eGFR and non-clear cell carcinoma correlated with the postoperative decreased renal function assessed by both ACE and PCE. The renal dysfunction also had influence in patients’ prognosis and we discovered that a cutoff value of 13.9 in ACE and 0.16 in PCE indicated a poor CSS respectively.

After following RN, the renal function of RCC patients significantly declined [22]. Yokoyama et al. [23] reviewed 341 Asian patients with RCC and found that the 3-year probability of eGFR> 60 mL/(min × 1.73 m^2) was 63%, while up to 11% patients suffered renal dysfunction of eGFR≤45 mL/(min × 1.73 m^2). Similarly, in another retrospective cohort study [24], the proportion was 65 and 36% respectively. In short-term renal function assessment, Tanaka et al. [25] demonstrated a 37% decreased in renal function of 155 patients in the following 2–4 weeks after RN. In one year after RN, the eGFR decreased by 27–36% [23, 26, 27]. In the present study, we observed a mean increase of 17.2% in serum creatinine and the chronic kidney disease (CKD) patients increased by 18.5% after RN with thrombectomy (29 vs. 42 CKD patients). What’s more, our finding in RN with thrombectomy was compatible with previous studies of pure RN, which results accorded with our sense. Table 2 and Table 3 further demonstrated that different TT levels were not associated with renal function change, which preliminary proved the irrelevance between TT and renal function. In the future, a cohort study of the comparison of postoperative renal function with and without TT needs to be made to further clarify this conclusion. Our study showed these data of perioperative renal function in order for clinicians to have a rough prediction preoperatively and make more active monitoring postoperatively.

After that, we also explore influencing factors of postoperative renal function, as well as its change rate. Preoperative eGFR seemed to be an important variable in assessing postoperative renal function, which was similar to previous studies. Lane et al. [28] reviewed 1169 patients with RN and concluded that low preoperative eGFR was associated with postoperative renal dysfunction. Krebs et al. [22] also suggested patients with preoperative poor renal function were at risk of postoperative end-stage kidney disease. This is well understood that postoperative renal function is related with preoperative one. What’s more, we divided the pathological features into two parts: clear cell carcinoma and non-clear cell carcinoma. According to the 2016 WHO classification [18], non-clear cell carcinoma consists of papillary renal cell carcinoma, nephroblastoma and Xp11.2 translocations/TFE3 gene fusion in our study. Interestingly, the present study also showed that the type of non-clear cell carcinoma was associated with greater renal function change, which indicated worse postoperative renal function. We assumed that different pathological types originated in specified histology, which might result in different effects on renal function. Mohammed et al. [29] found the histological types of RCCs arising in end-stage renal disease (ESRD) differed from that of sporadic RCCs. Moreover, Solomon et al. [30] studied a large cohort of RCC patients and found that declining renal function was independently related with an increased likelihood of papillary renal cell carcinoma histology, for the reason of protein expression with kidney injury. On the other hand, in Additional file 1: Table S1, we found the relationship between pathological histology and operation time, which was potentially associated with postoperative renal function. It was extremely hypothesized that for patients with normal preoperative renal function, the pathological type seemed more significant to indicate the decline of renal function postoperatively, considering that different biological characteristics, genetic mechanisms and clinicopathological variables in some aspect.

Several researches have been established to explore the prognostic outcomes after RN with thrombectomy. Tang et al. [31] carried on a median time of 45 months in follow-up and demonstrated that higher tumor thrombus level, lymph node and metastasis stages and adrenal gland invasion were the negative prognostic predictors significantly. However, previous studies did not include some useful index with renal function. Thus, we supplied previous studies and use ACE and PCE as predictive variables to make prognostic analysis, indicating that the ACE> 13.9 or PCE > 0.16 was significant risk factor in CSS. ACE and PCE seemed to be more accurate index in evaluate perioperative renal function and renal dysfunction. These results counseled our urologists that these patients had shorter survival time and more active monitoring of renal function should be done.

In our single institution experience, the reason of renal insufficiency after RN with thrombectomy consists of at least two aspects. One is renal ischemia caused by uninjured renal vein occlusion during operation. The other might be caused by insufficient circulation volume due to a large amount of bleeding during operation. In addition, the urine color and volume should be closely observed after operation. Several serum markers, such as serum creatinine, urea and electrolyte, should also be monitored. For patients with oliguria, increased serum creatinine or urea, it is recommended to supply blood volume and perform diuretic therapy in time, even making blood dialysis therapy if necessary.

Limitations of the study include that this is a retrospective and single institution study. In addition, lymph node was independent prognostic factor in univariate analysis, but not in multivariate analysis, as well as metastasis. These results might be attributed to the small sample size of the patients in our group patients, for the reason that this is a disease with a relatively low incidence and the number of patients who can be operated on is much less. In addition, the renal function is truly influenced by many factors. Our article only lists some of them, which is not comprehensive enough. However, there seems to be less studies about renal function changes in TT patients in our knowledge and our study has obtained some conclusions for foundation of further research. Therefore, next research needs lager cohort, more multi-center and prospective studies and we expect a new more complex index including all prognostic predictors in assessing the change of perioperative renal function of patients following RN with thrombectomy in the future.

## Conclusions

We explored several clinicopathological risk factors of predicting renal function change and their relationship with prognosis of RCC patients with tumor thrombus after nephrectomy and thrombectomy. The renal function change, which was associated with preoperative eGFR and pathological type, was prognostic risk factor for CSS and ACE> 13.9 indicated the worse prognosis.

## Supplementary information

**Additional file 1: Table S1.** Patient demographic and postoperative data according to different histology

## Data Availability

All the data used to support the findings of this study are currently under embargo while the research findings are commercialized. Requests for data, 6 months after publication of this article, will be considered by the corresponding author. The e-mail address: holmes_infinity@126.com (Shiying Tang).

## Abbreviations

ACE: Absolute change in estimated glomerular filtration rate
CKD: Chronic kidney disease
CN: Cytoreductive nephrectomy
CSS: Cancer-specific survival
CT: Computed tomography
eGFR: Estimated glomerular filtration rate
ESRD: End-stage renal disease
IVC: Inferior vena cava
MDRD: Modification of Diet in Renal Disease
MRI: Magnetic resonance imaging
OS: Overall survival
PCE: Percent change in estimated glomerular filtration rate
RCC: Renal cell carcinoma
RN: Radical nephrectomy
ROC: Receiver operating characteristic
TT: Tumor thrombus
WHO: World Health Organization
