# Supplementary material for: The effect of renal function change on renal cell carcinoma patients with tumor thrombus after nephrectomy and thrombectomy: a large Chinese center experience
Source: BMC Cancer. 2020 Jan 28;20:61. doi: 10.1186/s12885-020-6563-7 (PMC6986031; doi:10.1186/s12885-020-6563-7)
Supplement: Supplementary file 1 — Additional file 1: Table S1. Patient demographic and postoperative data according to different histology [file 12885_2020_6563_MOESM1_ESM.docx]

| Supplementary Table1. Patient demographic and postoperative data according to different histology | | | |
| --- | --- | --- | --- |
| Paprameters | Clear cell carcinoma | Non clear cell carcinoma | P value |
| Gender |  |  | 0.781 |
| Male | 85 | 16 |  |
| Female | 30 | 4 |  |
| Age, years | 59.8±9.5 | 56.0±16.4 | 0.594 |
| Body mass index, kg/m2 | 23.5±3.6 | 23.0±3.3 | 0.546 |
| ASA classification |  |  | **0.048** |
| 1 | 4 | 5 |  |
| 2 | 90 | 12 |  |
| 3 | 21 | 3 |  |
| Side |  |  | 0.38 |
| Left | 40 | 9 |  |
| Right | 75 | 11 |  |
| Tumor diameter, cm | 8.8±3.3 | 8.8±4.4 | 0.701 |
| Tumor thrombus grade |  |  | 0.283 |
| 0 | 29 | 2 |  |
| Ⅰ | 33 | 5 |  |
| Ⅱ | 33 | 6 |  |
| Ⅲ | 11 | 5 |  |
| Ⅳ | 9 | 2 |  |
| Preoperative serum creatinine, umol/L | 94.3±23.5 | 145.3±193.7 | 0.178 |
| Postoperative serum creatinine, umol/L | 125.6±129.2 | 108.9±62.1 | 0.586 |
| Preoperative eGFR, mL/(min×1.73m^2) | 74.9±21.4 | 67.9±23.6 | 0.364 |
| Postoperative eGFR, mL/(min×1.73m^2) | 68.4±24.1 | 76.0±30.9 | 0.316 |
| Operation approach |  |  | 0.639 |
| Laparoscopic approach | 64 | 10 |  |
| Open approach | 51 | 10 |  |
| Ipsilateral adrenalectomy |  |  | 0.899 |
| No | 50 | 9 |  |
| Yes | 65 | 11 |  |
| T stage |  |  |  |
| T3a | 26 | 1 |  |
| T3b | 68 | 15 |  |
| T3c | 5 | 2 |  |
| T4 | 16 | 2 |  |
| Lymph node dissection |  |  | 0.429 |
| No | 57 | 8 |  |
| Yes | 58 | 12 |  |
| Vascular wall invasion |  |  | 0.287 |
| No | 72 | 10 |  |
| Yes | 43 | 10 |  |
| Operation time, min | 323.9±119.9 | 411.9±114.2 | **0.003** |
| Intraoperative hemorrhage, ml | 1341.0±1625.2 | 1656.5±1744.9 | 0.197 |
| Lymph node metastasis |  |  | 0.177 |
| No | 108 | 17 |  |
| Yes | 7 | 3 |  |
| Metastasis or invasion of adrenal glands |  |  | 0.594 |
| No | 99 | 18 |  |
| Yes | 16 | 2 |  |
| Metastasis |  |  | 0.652 |
| No | 86 | 14 |  |
| Yes | 29 | 6 |  |
| Lymphatic vascular invasion |  |  | 0.287 |
| No | 72 | 10 |  |
| Yes | 43 | 10 |  |
| Perinephric tissues invasion |  |  | 0.178 |
| No | 84 | 10 |  |
| Yes | 33 | 8 |  |
| Renal pelvis invasion |  |  | 0.075 |
| No | 92 | 11 |  |
| Yes | 23 | 7 |  |
| Necrosis |  |  | 0.09 |
| No | 54 | 12 |  |
| Yes | 63 | 6 |  |
| Sarcomatoid differentiation |  |  | 0.305 |
| No | 96 | 17 |  |
| Yes | 21 | 1 |  |
| Postoperative complications |  |  | 0.069 |
| No | 71 | 8 |  |
| Yes | 44 | 12 |  |
| Serious complications |  |  | 0.724 |
| No | 30 | 8 |  |
| Yes | 15 | 3 |  |
| eGFR:estimated glomerular filtration rate,ISUP:International Society of Urological Pathology,ASA:American Society of Anesthesiologists | | | |
